# Neural tube development depends on notochord-derived sonic hedgehog released into the sclerotome

**DOI:** 10.1242/dev.183996

**Published:** 2020-05-26

**Authors:** Nitza Kahane, Chaya Kalcheim

**Affiliations:** Department of Medical Neurobiology, Institute of Medical Research Israel-Canada (IMRIC) and the Edmond and Lily Safra Center for Brain Sciences (ELSC), Hebrew University of Jerusalem-Hadassah Medical School, Jerusalem 9112102, P.O. Box 12272, Israel

**Keywords:** BMP, Dermomyotome, Dorso-ventral patterning, Hb9, Motoneurons, Myotome, Neural tube, Nkx, Paraxial mesoderm, Olig2, Pax7, Retinoic acid, Somite

## Abstract

Sonic hedgehog (Shh), produced in the notochord and floor plate, is necessary for both neural and mesodermal development. To reach the myotome, Shh has to traverse the sclerotome and a reduction of sclerotomal Shh affects myotome differentiation. By investigating loss and gain of Shh function, and floor-plate deletions, we report that sclerotomal Shh is also necessary for neural tube development. Reducing the amount of Shh in the sclerotome using a membrane-tethered hedgehog-interacting protein or Patched1, but not dominant active Patched, decreased the number of Olig2^+^ motoneuron progenitors and Hb9^+^ motoneurons without a significant effect on cell survival or proliferation. These effects were a specific and direct consequence of Shh reduction in the mesoderm. In addition, grafting notochords in a basal but not apical location, vis-à-vis the tube, profoundly affected motoneuron development, suggesting that initial ligand presentation occurs at the basal side of epithelia corresponding to the sclerotome-neural tube interface. Collectively, our results reveal that the sclerotome is a potential site of a Shh gradient that coordinates the development of mesodermal and neural progenitors.

## Abstract (precis)

**Summary:** Loss- and gain-of-function, and floor plate deletions, reveal that Shh that transits through the sclerotome is presented to the neuroepithelium from its basal aspect to affect motoneuron development.

## INTRODUCTION

The sonic hedgehog (Shh) protein plays fundamental roles in the development of the neural tube (NT) and somites (Borycki et al., 1998; Briscoe, 2009; Cairns et al., 2008; Ericson et al., 1997a,b; Gustafsson et al., 2002). Its signaling is initiated by binding of the ligand to the transmembrane receptor Patched (Ptc), that represses the pathway in its absence (Goodrich et al., 1997; Hidalgo and Ingham, 1990; Ingham et al., 1991; Johnson et al., 1996). Ligand binding to Ptc abrogates its repressive effect on Smoothened, a key effector essential for Hedgehog signal transduction (van den Heuvel and Ingham, 1996). The repressive role of Ptc correlates with its localization in the apical cilia, which function as a signal transduction compartment (Caspary et al., 2007; Rohatgi et al., 2007). Binding of Shh to Ptc removes the latter from the cilium, thereby allowing Smoothened to enter and propagate the signal further downstream (Milenkovic et al., 2009; Rohatgi et al., 2009) to regulate Gli activity (Briscoe and Thérond, 2013; Ribes and Briscoe, 2009).

Shh signaling is highly regulated by negative and positive modulators. Ptc1, Hedgehog interacting protein (Hhip) and Gli1 are direct targets of Shh, and the first two also inhibit its activity (Chuang and McMahon, 1999; Ingham and McMahon, 2001). Sulfatase1 (Dhoot et al., 2001), Boc, Gas and Cdo (Allen et al., 2011; Izzi et al., 2011) enhance ligand activities and are expressed in NT and/or developing mesoderm (Kahane et al., 2013).

Following neurulation, Shh secreted by the notochord (No) induces distinct ventral cell identities in the overlying NT by a mechanism that depends on relative concentrations and duration of exposure (Briscoe and Small, 2015; Dessaud et al., 2010; Stamataki et al., 2005). Moreover, the activity of Shh continues beyond this stage to regulate cell proliferation, survival and differentiation (Cayuso et al., 2006; Charrier et al., 2001). No-derived Shh is also involved in mesoderm patterning (Borycki et al., 1998; Cairns et al., 2008). A ventro-dorsal activity gradient of Shh/Gli signaling in the sclerotome was directly visualized using an *in vivo* reporter in mice (Kahane et al., 2013). In addition, in chick embryos, Shh spreads from the midline through the sclerotome to reach the dermomyotome (DM), in which it promotes terminal myogenic differentiation of DM-derived progenitors and maintains the epitheliality of DM cells (Kahane et al., 2013). Notably, in both the floor plate (FP) and the myotome, the activities of Shh are transient. This transient mechanism allows dynamic phase transitions to take place (Cruz et al., 2010; Kahane et al., 2013).

Because Shh is important for the development of both the NT and the mesoderm, two functionally interconnected systems, the question arises as to whether the effects of Shh on either tissue are independent of each other or interrelated. Furthermore, does the NT receive Shh directly from the producing sources (No and FP), or, given that the ligand is released into the mesoderm, can the latter serve as an ‘en passant’ pathway from which Shh affects aspects of both NT and mesoderm development? Answering these questions is of the utmost significance both for better understanding the mechanism of Shh activity and for achieving an integrated molecular view of regional development.

Here, we report that, in addition to affecting muscle development, reducing the amount of Shh in the sclerotome by Hhip1 or a membrane-tethered Hhip1 (Hhip:CD4) significantly reduces motoneuron numbers. The observed phenotypes are a specific and direct consequence of Shh depletion as they are rescued by excess Shh. Direct Shh targets are reduced and the effects of Shh are not mediated by other signaling pathways. Notably, the effects of Hhip:CD4 are phenocopied by the transmembrane receptor Ptch1 but not by PTC^Δloop2^, which does not recognize the ligand. In addition, by gain and loss of Shh function, and by FP deletions, we show that the sclerotome constitutes a dynamic substrate of No-derived Shh that acts both on motoneurons and on myotome development. Furthermore, grafting No fragments adjacent to the basal sclerotomal side of the NT profoundly affects its development compared with apical grafts. A similar basal grafting with respect to the DM significantly enhances myotome formation, suggesting a general need for initial ligand presentation at the basal side of epithelia. Together, our results uncover the sclerotome as a novel pathway through which No-derived Shh disperses to promote aspects of neural development.

## RESULTS

### Reduction of Shh in sclerotome by Hhip1 affects both myotome and motoneuron differentiation

To investigate possible Shh-mediated-interactions between neural and mesodermal progenitors, electroporations were performed in 23- to 25-somite stage (ss) embryos at the level of epithelial somites. This is the earliest timepoint at which the prospective sclerotome can be faithfully attained by focal electroporation. In this region, the NT is composed of proliferative cells (Kahane and Kalcheim, 1998) and neural patterning is already apparent and ongoing, as evidenced by the expression of *Nkx2.2*, *Olig2*, *Nkx6.2* and *Nkx6.1* (Fig. S1A-D). However, differentiation into Hb9-expressing motoneurons has not yet occurred at this stage (Fig. S1E) and only starts ∼10 h later at the level of somites 11-12 rostral to the last segmented somites (Fig. S1F). Hence, the timing of manipulations corresponds to the transition of proliferative progenitors undergoing specification into differentiated motoneurons (Ericson et al., 1996).

**Fig. 1.**
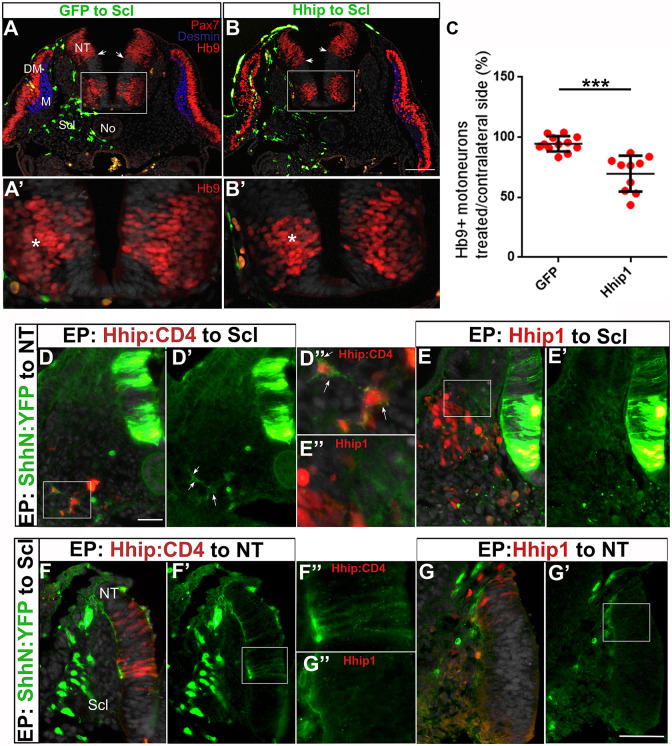
**Reduction of Shh in the sclerotome by Hhip1 affects both myotome and motoneuron differentiation.** (A,B) Electroporation of control GFP (A) or Hhip1/GFP (B) to the prospective sclerotome. One day later a reduction in myotome size (blue desmin staining) is apparent adjacent to the transfected cells. In addition, there is a ventral shift of the Pax7^+^ boundary in B (arrows). (A′,B′) Higher magnification of the insets in A,B, respectively, depicting a reduction of Hb9^+^ motoneurons in B upon Hhip1 treatment compared with the control side and with control GFP (A′). Asterisks denote motoneurons adjacent to electroporated sclerotomes. (C) Quantification of Hb9^+^ motoneurons in the hemi-NT facing the transfected mesoderm compared with control GFP (***P<0.001). Data are mean±s.e.m. *n*=12/treatment. (D-D″) Double electroporation of ShhN:YFP to the NT and Hhip1:CD4 to the sclerotome. Sclerotomal cells mis-expressing Hhip:CD4 (red) are decorated by Shh (green) immunolabeling (D′,D″, arrows). (E-E″) Double electroporation of ShhN:YFP to the NT and Hhip1 to the sclerotome. No labeling of Shh (green) is apparent in sclerotomal cells mis-expressing Hhip1 (red). D″,E″ are higher magnification images of the respective insets in D,E. (F-F″) Double electroporation of ShhN:YFP to the sclerotome and Hhip1:CD4 to the NT. The labeling of Shh (green) can be observed in both the basement membrane, as well as along neuroepithelial cells mis-expressing Hhip:CD4 (F′,F″). F″ is a higher magnification image of F′ in which only ShhN:YFP (green) is shown to highlight that Shh colocalizes with the red Hhip1:CD4-mis-expressing cells in the neuroepithelium (F). (G-G″) Double electroporation of ShhN:YFP to the sclerotome and Hhip1 to the NT. Labeling of Shh (green) in the basement membrane can be observed but there is no co-staining of neuroepithelial cells. G″ is a higher magnification image of the inset in G′. DM, dermomyotome; M, myotome; NT, neural tube; No, notochord; Scl, sclerotome. Scale bars: 50 µm.

**Fig. 2.**
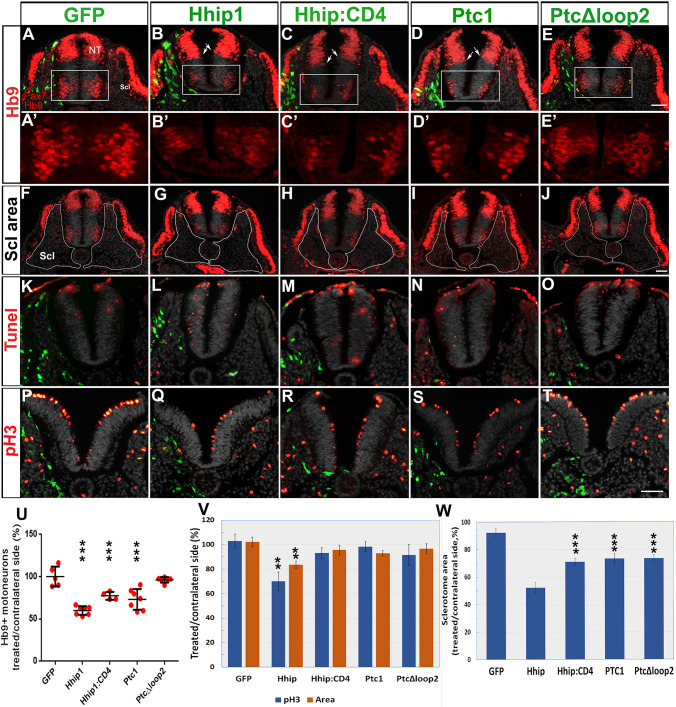
**Electroporation of Hhip:CD4 and Ptc1 into the sclerotome inhibit motoneuron development without significantly affecting progenitor proliferation or survival.** (A-E′) Electroporation (green) to the sclerotome followed by immunostaining for Pax7 and Hb9 1 day later. A′-E′ represent higher magnifications of the boxed regions in A-E. All plasmids, except PTC^Δloop2^, reduced the number of motoneurons adjacent to the transfected sclerotomes. Arrows in B-D show a slight ventralization of the ventral boundary of Pax7. (F-J) The same sections as in A-E in which the sclerotomes are delineated by white lines. (K-O) TUNEL staining (red). Weak immunostaining of electroporated plasmids (green) can be observed because no anti-GFP antibodies were used to enable better visualization of apoptotic nuclei. Only Hhip1 resulted in numerous TUNEL^+^ nuclei adjacent to the transfected sclerotome. Ectodermal staining reflects non-specific reactivity. (P-T) staining of mitotic nuclei with anti-pH3 (red). (U) Quantification of motoneurons in the hemi-NT facing the transfected mesoderm compared with control GFP. (V) Quantification of mitotic nuclei and area in the hemi-NT adjacent to the electroporated sclerotome. (W) Quantification of the relative area of electroporated sclerotomes based on sections such as those shown in F-J. NT, neural tube; Scl, sclerotome. Data are mean±s.e.m. **P<0.03, ***P<0.01. Scale bars: 50 µm.

Previously, we reported that the traversing of the sclerotome by Shh is necessary for myotome differentiation, as misexpression of the high-affinity Shh antagonist Hhip1 in the sclerotome resulted in smaller myotomes expressing desmin accompanied by a corresponding accumulation of Pax7^+^ progenitors (Kahane et al., 2013) (Fig. 1A,B). Here, we report that the hemi-NT facing the transfected mesoderm also exhibited a 40% reduction in the number of Hb9^+^ motoneurons compared with control GFP (Fig. 1A′,B′,C, *P*<0.001, *n*=12/treatment). This is a significant effect given that electroporation is a mosaic technique that attains only a fraction of cells, thus causing ligand reduction rather than a total depletion. Moreover, the ventral boundary of Pax7 expression was frequently shifted ventrally (Fig. 1A,B), probably owing to the smaller ventral extent of the transfected hemi-NT (Fig. 2).

These results can be explained by the sclerotome constituting a substrate through which Shh disperses and from which the ligand is provided to both mesodermal and neural progenitors. Alternatively, they might result from Hhip1 moving from the transfected mesoderm towards the NT. Indeed, in spite of initial findings showing that Hhip1 is a transmembrane glycoprotein with cell-autonomous functions (Bishop et al., 2009; Chuang and McMahon, 1999), it has been reported that Hhip1 is secreted and able to exert long-range effects on Shh signaling (Holtz et al., 2015; Kwong et al., 2014).

### Differential behavior of secreted Hhip1 compared with membrane-tethered Hhip1:CD4 vis-à-vis Shh

To distinguish between the above possibilities, we produced a Hhip:CD4 plasmid encoding membrane-tethered Hhip1, which is unable to undergo secretion (Holtz et al., 2015; Kwong et al., 2014). First, we investigated whether NT or sclerotomal cells misexpressing either Hhip1 or Hhip:CD4 can sequester Shh. To this end, we used a plasmid encoding the N-terminus of Shh fused to YFP (ShhN:YFP). ShhN:YFP is able to undergo palmitoylation but not the addition of cholesterol, a property that enables free movement of the mutant protein when compared with native Shh (Beug et al., 2011). When electroporated to the sclerotome, secreted ShhN:YFP was apparent along the basement membrane of the NT where it colocalized with laminin, yet no fluorescent signal was detected in neuroepithelial cells (Fig. S2A-C).

When double electroporations of ShhN:YFP to the NT and Hhip:CD4 to the sclerotome, or vice versa, were performed, the NT progenitors or Hhip:CD4-transfected sclerotome, respectively, were decorated with ShhN:YFP, demonstrating that Hhip:CD4 binds and immobilizes the ligand on the surface of the expressing cells (Fig. 1D-D″,F-F″). In contrast, no such colocalization could be observed in either the sclerotome or the NT upon electroporation of ShhN:YFP and Hhip1 (Fig. 1E-E″,G-G″), which was consistent with the notion that native Hhip1 is a secreted protein.

In addition, we monitored the expression of endogenous Shh protein upon transfection of Hhip1 or Hhip:CD4 to the sclerotome. Shh protein was evident intracellularly as well as being associated with the cell membranes of the FP and No, in which it was probably exposed to the external membrane surface (Fig. S2D-F′). Electroporation of control GFP and of Hhip:CD4 had no effect on Shh immunoreactive protein in either the No or FP (Fig. S2D-E′). In contrast, misexpression of Hhip1 markedly reduced Shh levels in both structures unilaterally adjacent to the transfected cells; this reduction was mainly apparent at the basal sides of these structures, closer to the transfected Hhip1 (Fig. S2). This effect may result from Hhip1 masking antibody binding to the ligand, as the 5E1 Shh antibody and Hhip1 bind to the same pseudo-active site on the Shh molecule (Bishop et al., 2009; Maun et al., 2010). Together, these data confirm that both Hhip and Hhip:CD4 bind Shh but, in contrast to Hhip:CD4, Hhip1 is secreted to adsorb Shh at a distance.

### The effects of Hhip:CD4 in the NT resemble those observed with other Shh inhibitors

Next, we examined the NT to check the specificity of Hhip:CD4 relative to other known inhibitors of Shh. Electroporation of Hhip:CD4, like that of Hhip1, Ptc1 or PTC^Δloop2^ to hemi-NTs, significantly reduced the number of Hb9^+^ motoneurons and that of pH3^+^ mitotic nuclei while enhancing cellular apoptosis. Furthermore, the total area of the transfected hemi-NTs, which reflects overall changes in cell number (see Materials and Methods), was significantly smaller in all treatments when compared with controls (Fig. S3, *n*=4/treatment, *P<0.05, **P<0.03, ***P<0.01). Thus, these effects on motoneurons could result from reduced cell differentiation or, indirectly, from effects on progenitor proliferation or survival. These data confirm that Shh acts both as a mitogen and survival factor (Cayuso et al., 2006; Charrier et al., 2001). Most importantly, they demonstrate that Hhip:CD4, by acting like Hhip1, Ptc1 or PTC^Δloop2^, is a specific tool with which to abrogate Shh activity.

### Local depletion of Shh activity by Hhip:CD4 or Ptc1 in the sclerotome inhibits motoneuron specification and/or differentiation in the NT, yet has no significant effect on cell proliferation or survival

Next, we investigated whether the effects originally observed across tissues (e.g. between the NT and mesoderm) with secreted Hhip1 (Fig. 1) can be mimicked by the misexpression of two different Shh inhibitors, Hhip:CD4 and the Shh receptor Ptc1, both of which are membrane-associated molecules.

First, we confirmed that electroporation of Hhip1 to the sclerotome caused significant effects in the NT, as expected from a secreted molecule. These included: a reduction in the number of Hb9^+^ motoneurons (Fig. 2B,B′,U; *n*=8, *P*<0.01); a decreased number of pH3^+^ mitotic nuclei when measured in the entire hemi-NT (Fig. 2Q,V; *n*=4, *P*<0.03) but not when the ventral domain-containing motoneurons were considered (94.2±4% versus 98.4±6% in Hhip1 and GFP, respectively); enhanced apoptosis (Fig. 2L; *n*=4); and an overall decrease in the area of the respective hemi-NT (Fig. 2V; *n*=4, *P*<0.03) compared with controls (*n*=5, Fig. 2A,A′,K,P,V).

Transfection of both Hhip:CD4 and Ptc1 also significantly affected motoneuron numbers (*n*=38, *P*<0.01 and *n*=7, *P*<0.01, respectively, Fig. 2C,C′,D,D′,U), yet had no significant effect on overall cell proliferation (Fig. 2V; *n*=4 and 7), on the proliferation of ventral progenitors (113±10%, 110±14% versus 98.4±6% for Hhip:CD4, Ptc1 and GFP) or on total hemi-NT area (*n*=4 and 6, respectively) opposite the treated sclerotomes (Fig. 2R,S,V).

**Fig. 3.**
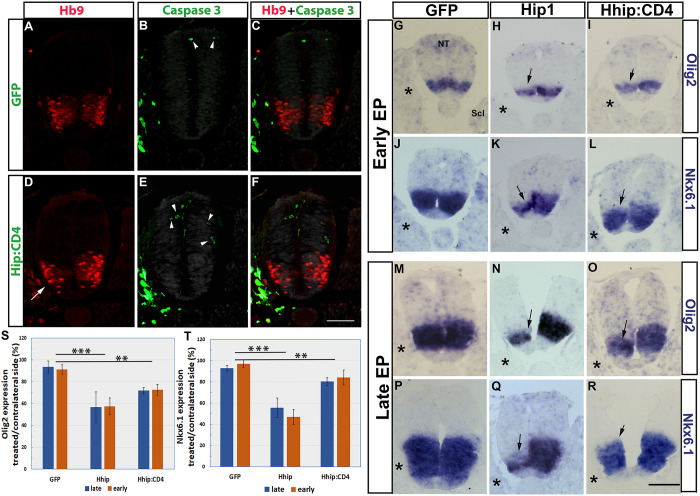
**Electroporation of Hhip:CD4 into sclerotome reduces the extent of *Olig2* and *Nkx.6.1* expression in NT without affecting cell survival.** (A-F) Electroporation of control GFP (A-C) or Hhip:CD4 (D-F) (green cells in the sclerotome). A unilateral reduction of Hb9^+^ motoneurons adjacent to the transfected sclerotome (arrow in D) can be observed. Green cells in the NT represent caspase 3^+^ nuclei (arrowheads). Only a few apoptotic nuclei are evident in both control and treated embryos primarily localized to the dorsal NT but not to the motoneuron area. See Results section for quantification. (G-R) Electroporation of control GFP, Hhip1 or Hhip:CD4. G-L represent early electroporations; M-R show late electroporations. Asterisks denote transfected sclerotomes. Arrows mark mRNA expression on the experimental side. The extent and/or intensity of mRNA expression were reduced in Hhip1/Hhip:CD4-treated embryos. (S,T) Quantification of the relative expression of *Olig2* and *Nkx6.1* upon early versus late electroporations. **P<0.01, ***P<0.001. NT, neural tube; Scl, sclerotome. Scale bar: 50 µm.

Likewise, cell death, as measured by TUNEL staining was not qualitatively affected (Fig. 2M,N, *n*=4 and 6). The number of apoptotic nuclei was quantified using caspase 3 immunostaining upon control GFP or Hhip:CD4 electroporation to the sclerotome. A very low number of positive cells and no measurable difference in the ratio of caspase 3^+^ cells were detected between both treatments (control GFP, 2.3±0.47 and 2.3±0.7 cells/section; and for Hhip:CD4, 1.7±0.5 and 1.2±0.5 cells/section opposite the treated and contralateral sides, respectively; *n*=4/treatment, Fig. 3A-F). Most caspase 3^+^ apoptotic nuclei were located in the dorsal half of the NT and the amount of caspase 3^+^ nuclei in the Hb9^+^ domain was negligible. In contrast, a clear reduction in Hb9^+^ MNs was observed in the same sections upon Hhip:CD4 electroporation to the sclerotome (Fig. 3D-F).

Thus, reduction of Shh activity in the mesoderm by either Hhip:CD4 or Ptc1 mainly affects motoneuron differentiation, contrasting with Shh abrogation in the NT where all parameters were considerably compromised (Fig. S3). This suggests that motoneuron differentiation might be more sensitive to a reduced amount of ligand and further indicates that progenitor proliferation, survival and motoneuron differentiation might be separable processes that depend upon different Shh concentrations.

As a control for Ptc1 activity, we electroporated PTC^Δloop2^, which is unable to bind circulating Shh and acts cell autonomously to inhibit its signaling. PTC^Δloop2^, as with Hhip1, Hhip:CD4 and Ptc1, adversely affected the size of the electroporated sclerotomes when compared with the intact contralateral ones (Fig. 2F-J,W; *n*=5, *P*<0.01), altogether demonstrating that the traversing of the sclerotome by Shh is necessary for proliferation and/or survival of these mesodermal progenitors. In contrast, PTC^Δloop2^ had no significant effect on proliferation, survival or the total area of adjacent NT cells (Fig. 2O,T,V), neither did it affect proliferation of motoneuron precursors (111±13% versus 98.4±6% for PTC^Δloop2^ compared with GFP). As expected, unlike Ptc1, PTC^Δloop2^ had no effect on motoneurons (Fig. 2E,E′,U), suggesting that reduced sclerotomal size is not sufficient to account for the observed loss of motoneurons.

It is worth mentioning that Hhip1, Hhip:CD4 and Ptc1 also caused a slight ventralization of the ventral boundary of Pax7 expression (arrows in Fig. 2B-D), which was not apparent upon treatment with PTC^Δloop2^ (Fig. 2E).

The possibility that Hhip:CD4 or Hhip1 affect specification of NT cells was further tested by examining the expression of *Olig2*, a marker of motoneuron progenitors and of *Nkx6.1*, which also extends more ventrally. Transfections were performed at either 12 ss (early) or at 25 ss (late), and embryos were fixed 14 h later.

Expression of *Olig2* and *Nkx6.1* was significantly reduced adjacent to the electroporated sclerotomes of both Hhip1- and Hip:CD4-treated embryos. Moreover, no difference in the extent of the effects was monitored in early versus late electroporations, yet Hhip1 exhibited a stronger phenotype than Hhip:CD4 at either stage (Fig. 3G-T, *n*=5/treatment), consistent with the former being soluble and, therefore, more efficient in ligand sequestration. This suggests that depletion of Shh in the sclerotome also affects the ongoing specification of motoneuron progenitors, a process that had already begun before the electroporations were performed (Fig. S1).

**Fig. 4.**
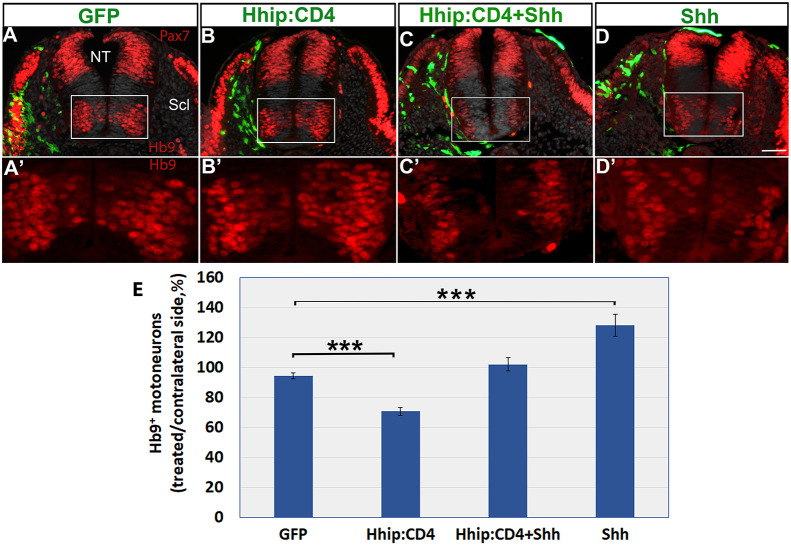
**Depletion of Shh activity by Hhip:CD4 in the sclerotome inhibits motoneuron development and is rescued by co-treatment with Shh.** (A-D) Electroporation of the depicted plasmids to the sclerotome (Scl) (green). Hhip:CD4 to Scl reduces motoneuron numbers compared with control GFP, whereas co-transfection with Shh rescues the effect. A′-D′ are higher magnification images of the insets in A-D. (E) Quantification of Hb9^+^ motoneurons. Data are mean±s.e.m. ***P<0.001. NT, neural tube. Scale bar: 50 µm.

We further assessed the specificity of Hhip:CD4. Although sclerotomal missexpression of Hhip:CD4 significantly affected the number of Hb9^+^ cells, co-treatment of Hhip:CD4 with Shh rescued the effect back to control levels, further suggesting that Hhip:CD4 abrogates Shh activity. Moreover, Shh alone significantly enhanced motoneuron differentiation (Fig. 4A-E; *n*=12, 26, 8 and 16, for control GFP, Hhip:CD4, Hhip:CD4+Shh and Shh alone, respectively, *P*<0.001).

### Local depletion of Shh activity by Hhip:CD4 or Ptc1 in NT inhibits myotome differentiation in the mesoderm

**Fig. 5.**
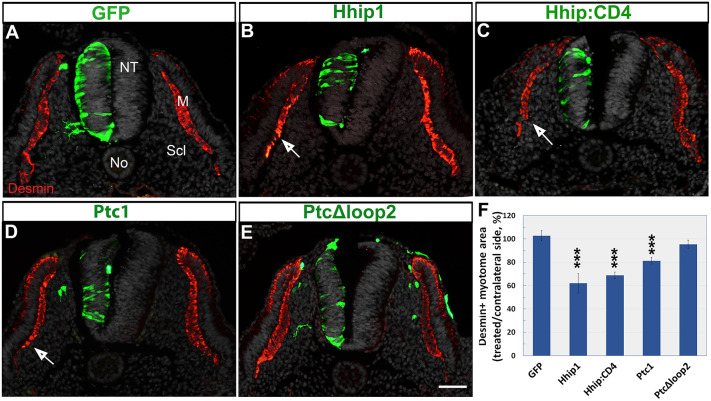
**Electroporations of Hhip, Hhip:CD4 or Ptc1, but not of PTC^Δloop2^, into the NT reduce myotome size.** (A-E) Electroporations of the depicted plasmids (green). Hhip1, Hhip:CD4 and Ptc1 reduce the size of adjacent desmin^+^ myotomes (arrows, red) compared with control GFP yet PTC^Δloop2^ has no significant effect. (F) Quantification of myotome size. ***P<0.001. Scale bar: 50 µm.

Next, we examined whether the reduction of Shh in the NT influences myotomal size, measured as the area of desmin^+^ immunoreactivity. Electroporation of control GFP had no effect on myotome size (*n*=14), whereas Hhip1 and Hhip:CD4 caused a significant decrease in myotomal size adjacent to the transfected side (Fig. 5A-C,F; *n*=3 and 10, respectively, *P*<0.001). Electroporation of Ptc1 exhibited a similar effect (*n*=16, *P*<0.001); in contrast, PTC^Δloop2^ revealed no reduction (Fig. 5D-F; *n*=6).

Together, our data suggest that reducing the amount of Shh circulating through the sclerotome promotes a concomitant loss of effective Shh in the NT and vice versa. As Hhip:CD4 and Ptc1 are membrane bound, and not secreted, the present results could be explained by the sclerotome, which is a substrate for Shh dispersal, representing a common pool that supplies the ligand to both tissues. Consistent with this notion, sclerotomal Shh is not likely to act by affecting ligand levels in the producing cells because the inhibition of Shh by Hhip:CD4 in the sclerotome had no effect on Shh expression in either FP or No (Fig. S2E,E′).

### The effects of Hhip:CD4 are a direct consequence of Shh depletion

**Fig. 6.**
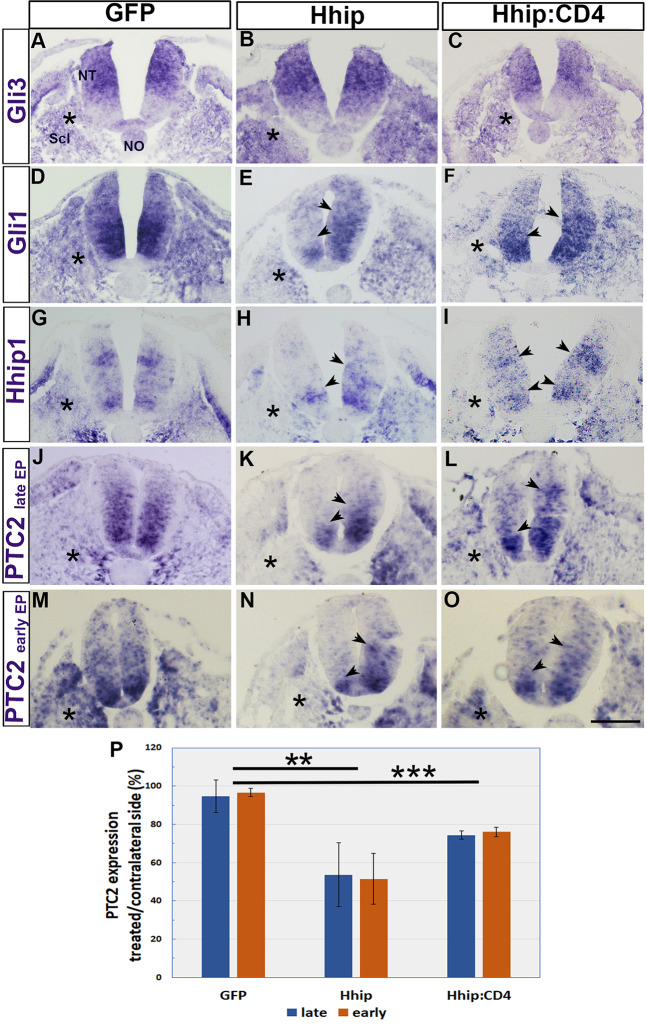
**The effects of Hhip1 and Hhip:CD4 on the NT are a direct consequence of Shh depletion.** (A-I) *In situ* hybridization for *Gli3*, *Gli1* and *Hhip1* following electroporation of the depicted plasmids. (J-O) *In situ* hybridization for *Ptc2* following late or early electroporations of control GFP, Hhip1 or Hhip:CD4. Asterisks mark the electroporated sites. Control GFP had no effect on the bilateral expression of any of the genes in the NT. In contrast, Hhip1 and Hhip:CD4 transfected to the sclerotome reduced *Gli1*, *Hhip1* and *Ptc2* mRNAs unilaterally (D-O, arrowheads) but not *Gli3* mRNA (A-C). (P) Quantification of the relative expression of *Ptc2* in treated/contralateral sides of late and early electroporations (*n*=5 embryos/treatment, **P<0.01, ***P<0.001). NT, neural tube; No, notochord; Scl, sclerotome. Scale bar: 50 µm.

**Fig. 7.**
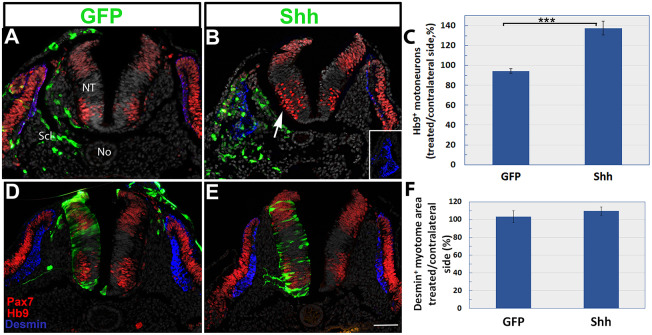
**Gain of Shh function** **in the sclerotome enhances motoneuron differentiation but Shh misexpression in the NT has no effect on the myotome.** (A-C) Electroporation of Shh (green) to the sclerotome (Scl) enhances the number of Hb9^+^ motoneurons compared with the control (arrow in B, red). Quantification in C, ***P<0.001. (D-F) Electroporation of Shh (green) to the NT has no effect on the size of desmin^+^ myotomes (blue) or on the expression of Pax7 (red) in the DM. Quantification in F. Data are mean±s.e.m. No, notochord; NT, neural tube; Scl, sclerotome. Scale bar: 50 µm.

The similarity between the effects of Hhip:CD4 and Ptc1 (Fig. 2), and the rescue of motoneurons by co-electroporation of Shh and Hhip:CD4 (Fig. 4) suggests that the effects of Hhip1 and Hhip:CD4 are specifically mediated by ligand depletion. To further investigate the possibility of direct versus indirect effects, we examined the expression of three transcriptional targets of Shh, *Gli1*, *Hhip1* and *Ptc2*, and compared it with *Gli3* mRNA expression, which is not directly regulated by Shh (Sasaki et al., 1999). Electroporation of control GFP, Hhip1 or Hhip:CD4 to the sclerotome had no effect on *Gli3* mRNA in either the NT or the sclerotome (Fig. 6A-C). In contrast, transfections of Hhip1 or Hhip:CD4 to the sclerotome reduced *Gli1*, *Hhip1* and *Ptc2* mRNAs in the adjacent hemi-NT, as well as in the transfected sclerotomes, compared with contralateral sides and with control GFP (Fig. 7D-O). *Ptc2* expression was further quantified in embryos electroporated at either 25 ss (late) or 12 ss (early); both revealed a significant reduction compared with control GFP but no difference between the stages was observed (Fig. 6J-P; *n*=5, **P<0.01, ***P<0.001). Notably, although the electroporation of both plasmids resulted in visible reductions, the phenotypes obtained with soluble Hhip1 were more striking than those with Hhip:CD4, consistent with a stronger effect of the former on motoneuron numbers and on *Olig2* expression (Fig. 1C; Fig. 2V; Fig. 3). Thus, the observed effects specifically and directly result from ligand reduction.

Next, we examined the possibility of secondary effects across tissues mediated by Shh depletion. Two candidates are BMP, which acts antagonistically to Shh (Dessaud et al., 2007; Ericson et al., 1997a; McMahon et al., 1998), and retinoic acid from the somite that affects NT development (Diez del Corral et al., 2003; Wilson et al., 2003). To this end, control GFP or Hhip:CD4 were electroporated into the sclerotome. If the effects on the NT of Shh deprivation in the sclerotome are mediated by BMP, it is predicted that the dorsal extent and intensity of BMP signaling will be expanded and/or increased, respectively. No changes in the area or intensity of pSmad 1,5,8 immunoreactivity, a readout of BMP activity restricted to the dorsal NT, were measured upon Hhip:CD4 treatment (Fig. S4, *n*=4 per treatment). As an internal control, we observed a reduced number of Hb9^+^ motoneurons in the ventral NT adjacent to the transfected side (Fig. S4B).

In addition, control GFP or Hhip:CD4 were electroporated into the sclerotome and retinoic acid response element-alkaline phosphatase (RARE-AP), a specific reporter of retinoic acid activity, was co-electroporated into the NT along with GFP to monitor electroporation efficiency. The specificity of RARE-AP was first tested by co-electroporating it with a dominant-negative receptor plasmid that abolished RARE-AP signal (Fig. S5A-B″). No measurable effect in the relative intensity of RARE-AP compared with GFP was observed in the NT upon reduction of Shh in the neighboring sclerotome when compared with the control (*n*=9/treatment, Fig. S5C-E).

Together, these results show that the observed effects are a direct consequence of Shh depletion. Thus, the sequestration of Shh in the sclerotome by Hhip1, Hip:CD4 or Ptc1 results in a corresponding reduction of Shh ligand in the NT, and vice versa.

### Gain of Shh function in the sclerotome enhances motoneuron specification and/or differentiation but Shh misexpression in the NT has no effect on the myotome

Our loss-of-function results are consistent with the possibility that Shh can translocate bidirectionally between the mesoderm and the NT. To gain additional insight into the directionality of Shh effects across the NT and the mesoderm, we adopted a complementary gain-of-function approach. Control GFP or full-length Shh were electroporated into the sclerotome. An increase of 30-40% in the number of Hb9^+^ motoneurons was observed 1 day later in the NT of Shh-treated embryos ipsilateral to the treated side compared with controls (*P*<0.001, *n*=9/treatment, Fig. 7A-C; Fig. 4E).

Reciprocally, control GFP or full-length Shh were electroporated into hemi-NTs, and the size of adjacent desmin^+^ myotomes was examined. Although the overall size of the transfected hemi-NT increased, no significant change in myotomal size was observed and there were no apparent effects on the DM or the sclerotome (*n*=6 and 5 in control and treated embryos, respectively, Fig. 7D-F).

Our gain-of-function data are, therefore, inconsistent with the simpler possibility, based solely on data from loss of Shh activity, that Shh moves bidirectionally between the mesoderm and the NT. Whereas excess Shh in the mesoderm profoundly affects NT development, the observation that gain of Shh in the NT has no effect on myotome development is in line with Shh being transported into the neuroepithelium but not outside into the mesoderm. In this regard, our finding that Shh depletion in the NT inhibits myotome differentiation (Fig. 5) is consistent with this procedure causing a corresponding lower effective concentration in the mesoderm. This could be accounted for by enhanced uptake of the ligand into the NT cells via directional baso-apical transport from the sclerotome. This suggests that the sclerotome constitutes a common substrate of No-derived Shh that serves both tissues.

### Sclerotome- but not FP-derived Shh is necessary for motoneuron specification and/or differentiation

The accepted view suggests that the development of various NT cell types depends upon a local ventrodorsal gradient of Shh directly emanating from the No and/or FP (Smith, 1993; Trousse et al., 1995). Our present results show that the sclerotomal pool of Shh, originally released from No/FP, is also necessary for motoneuron development (Fig. 5,6). Hence, we examined the relative contribution of the FP compared with sclerotomal Shh to the development of Hb9^+^ neurons.

**Fig. 8.**
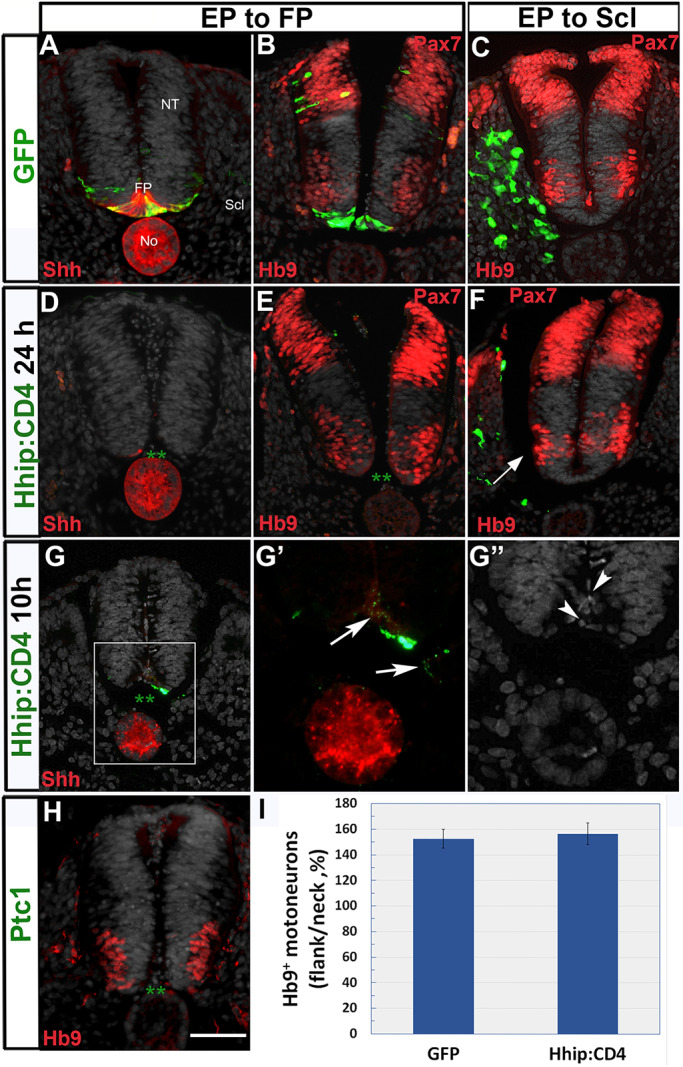
**Sclerotome-derived Shh, but not FP-derived Shh, is necessary for motoneuron development.** (A,B) Dorsoventral electroporation of control GFP showing (A) the presence of the labeled FP that co-expresses Shh protein. (B) Hb9^+^ motoneurons are dorsal to the labeled FP. (C) Hb9^+^ motoneurons in control GFP electroporation to the sclerotome. (D,E) Loss of FP tissue 24 h post-Hhip:CD4 electroporation. Asterisks in D,E denote the absence of a FP; D shows concomitant absence of Shh expression. Hhip:CD4 electroporation has no effect on ventral Hb9^+^ motoneurons or dorsal Pax7 expression (both in red). (F) In contrast, fewer motoneurons are apparent adjacent to the sclerotome transfected with Hhip:CD4 (arrow). (G-G″) Disintegration of the FP 10 h following Hhip:CD4 transfection. Note in G and G′ the almost complete absence of GFP signal in the FP domain (with only one GFP^+^ cell remaining), as well as the punctate expression of GFP (arrows) corresponding to disorganized and pyknotic nuclei (arrowheads in G″). (H) Loss of the FP upon electroporation of Ptc1 (green asterisks indicate the lack of an FP) shows no apparent effect on motoneurons. (I) Quantification of the proportion of Hb9^+^ motoneurons in flank (electroporated) and neck (intact region). Data are mean±s.e.m. No, notochord; NT, neural tube. Scale bar: 50 µm.

Control GFP or Hhip:CD4 were electroporated dorsoventrally to attain the FP. Control GFP had no effect on FP integrity or on Shh immunoreactivity (*n*=7, Fig. 8A). Surprisingly, by 24 h, electroporation of Hhip:CD4 resulted in the total absence of the FP and consequent loss of FP-derived Shh (*n*=10, Fig. 8D,E). Notably, at ≤10 h post-electroporation, the beginning of FP disintegration, which is reflected by the presence of pyknotic nuclei, loss of Shh and the punctate appearance of transfected Hhip:CD4-GFP were observed (Fig. 8G-G″, *n*=5). This early loss of FP enabled us to accurately monitor its contribution to motoneuron development. Because the effect is bilateral, in order to obtain a reliable measurement, the proportion of Hb9^+^ neurons was measured as the ratio between neurons in the flank lacking a FP to the intact neck of the same embryos. In spite of FP disappearance, the proportion of flank-level motoneurons was unaltered when compared with control embryos that had received GFP (*n*=4/treatment, Fig. 8B,E,I). To further examine the need for the FP, control GFP and Hhip:CD4-treated embryos were fixed 40 h following transfection to the brachial level. As shown in Fig. S6, no FP remained after electroporation with Hhip:CD4, which was revealed by a lack of GFP signal (Fig. S6A-E), by disappearance of Shh mRNA, the latter still apparent in the No (Fig. S6J,L), and by nuclear Hoechst staining (Fig. S6D,D′,H,H′,K,K′,M,M′). Nevertheless, no apparent reduction in motoneurons was detected in treated versus control GFP cases (Fig. S6A-C,E-G), and the proportion of Hb9^+^ motoneurons compared between caudal brachial (electroporated) and rostral brachial (intact) levels did not significantly change (*n*=5/treatment, Fig. S6A-I). Likewise, expression of *Olig2* mRNA, which was visible dorsal to the strong *Shh* mRNA signal in the FP of controls (Fig. S6J,K), remained similar in Hhip:CD4-treated embryos that lacked a FP (*n*=6/treatment). Notably, as shown in Fig. S6L-M′, cells sometimes filled the gap at the ventral midline into which *Olig2* expression extended. In such cases, nuclear organization was diffuse compared with the ordered basal location of nuclei in FP-containing NTs (Fig. S6D′,K′,M′).

Similar to what was observed with Hhip:CD4, electroporation of Ptc1 also compromised FP integrity while having no apparent effect on motoneurons (*n*=5, Fig. 8H). In contrast, inhibition of Shh in the sclerotome by Hhip:CD4 exhibited a visible reduction in motoneurons (Fig. 8C,F; Fig. 2; Fig. 3). Thus, we conclude that the traversing of the sclerotome by Shh plays a significant part in motoneuron differentiation, whereas the direct supply of ligand by FP has no apparent contribution, at least not at the stages examined here.

### A basal, but not apical, presentation of Shh is required for ligand activity on both the NT and the DM/myotome

**Fig. 9.**
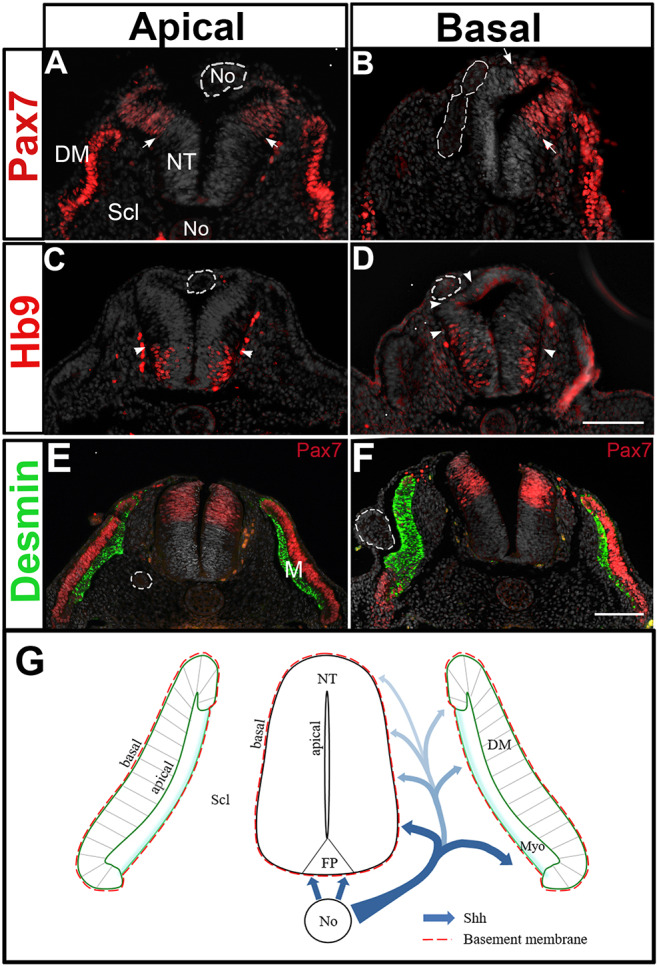
**A basal, but not apical, presentation of Shh is required for ligand activity on both the NT and the DM/myotome.** (A-F) A model accounting for the effects of Shh traversing the sclerotome on both NT and myotome development. Implantation of No fragments (dashed circles) in apical (A,C,E) or basal (B,D,F) positions vis à vis the dorsal NT or the DM (E,F). (A,C) A No piece was grafted inside the NT facing its luminal side. One day later it localizes in the dorsal NT. No significant effect on Pax7 (arrows) or Hb9^+^ motoneurons (arrowheads) is apparent. (B,D) A No piece was grafted in a basal position vis à vis the dorsal NT. A dorsal restriction of Pax7 (arrows in B), as well as a dorsalward expansion of Hb9^+^ motoneurons (arrowheads in D). (E,F) A No fragment localized apical to the DM causes a mild change in the adjacent desmin^+^ myotome (green, E), whereas the basally located No strongly increases myotomal size and promotes an *in situ* differentiation of the DM into the myotome with concomitant loss of Pax7 expression. (G) A proposed model for the activity of No-derived Shh. The No secretes Shh that acts on the ventral NT and also traverses the sclerotome (Scl), which is both a pathway for ligand movement and also a target of its activity. We propose that a ventral to dorsal gradient of ligand is created in the sclerotome and plays a pivotal role both on the myotome as well as on motoneuron development. Shh is thus presented to the target epithelial cells via its basal domain, probably by initial association with the laminin-containing basement membrane. Scale bars: 50 μm.

As the transit of Shh through the sclerotome is needed for NT development, we predicted that the NT would be more sensitive to Shh presented from its basal side abutting the sclerotome than from its apical side. To examine this hypothesis, fragments of No were grafted into either the lumen of the NT (apical) or between somites and the NT (basal grafts). After 1 day, there was no apparent reduction in the extent of Pax7 expression in the luminal grafts; and only a mild reduction in the intensity of Pax7 was observed (Fig. 9A,B). In contrast, Pax7 was dorsally restricted when facing the basal grafts (Fig. 7B). The latter also caused a characteristic bending of the NT, previously reported to represent an ectopic FP-like structure (van Straaten et al., 1988) (*n*=6 and 6 for apical versus basal grafts, Fig. 9B,D). In addition, the amount of Hb9^+^ motoneurons was unchanged by the apical grafts yet was markedly increased in the basal grafts in which the Nos were similarly localized in a dorsal position vis-à-vis the NT (*n*=6 and 6 for apical versus basal grafts, Fig. 9C,D), in agreement with classical No graft experiments (van Straaten et al., 1989).

Likewise, the implantation of No fragments at epithelial somite levels, and at an apical position with respect to the DM, had only a mild effect on the subsequent development of desmin^+^ myotomes, with no apparent alteration in Pax7 expression in the DM. In striking contrast, equivalent grafts performed basally to the prospective DM, produced large myotomes expressing desmin and a radical *in situ* differentiation of the DM into muscle at the expense of Pax7^+^ progenitors (*n*=5 and 7 for apical versus basal grafts, Fig. 9E,F). Therefore, an initial basal presentation of the ligand, vis-à-vis the target epithelium, is required for the activity of Shh. This is consistent with our results showing that Shh emanating from the sclerotome and reaching the NT from its basal side is necessary and sufficient for aspects of NT differentiation.

## DISCUSSION

We uncover a previously unknown domain, the sclerotome, as being an important ‘en passant’ substrate of Shh that influences not only DM and myotome development, as previously shown (Kahane et al., 2013), but also aspects of NT development, such as specification and differentiation of motoneurons (Fig. 9G). Loss-of-function data show that local depletion of Shh in either the NT or the sclerotome results in major defects across tissues, such as reduced myotomal size and fewer motoneurons. Reciprocally, only gain of Shh function in the sclerotome enhances motoneuron differentiation, whereas misexpression of the ligand in the NT has no effect on myotomal size. Together, these results suggest that Shh specifically secreted into the sclerotome constitutes a common pool that supplies both tissues.

In contrast to the observed reduction in motoneurons upon inhibition of Shh in the sclerotome, ablation of the FP has no short-term effects on motoneurons. In addition, embryos without a FP, fixed 2 days after electroporation, did not exhibit a significant reduction in *Olig2* or *Hb9*. This is consistent with the development of a normal ventral pattern in *Gli2* mutants lacking a FP (Chamberlain et al., 2008; Matise et al., 1998). Likewise, loss of Shh in FP did not dramatically alter ventral neural patterning yet altered later gliogenesis (Yu et al., 2013). Furthermore, the abrogation of Shh in the FP under the regulation of Brn4 revealed normal short-term expression of *Nkx2*.*2* and *Olig2* but a reduction at later stages (Dessaud et al., 2010). Although this initial phenotype is consistent with our results, we did not observe a later effect in avian embryos when electroporation was performed at the epithelial somite stage. Perhaps an even earlier deletion of the FP, which is technically challenging in the avian embryo, would have resulted in effects similar to those obtained in mice. The above results indicate that Shh, which is required at this stage for motoneuron development, does not have to emanate from direct contact with the FP, which is an integral part of the neuroepithelium, and any basal source of ligand is sufficient. It is therefore possible that, in the absence of a FP, the No compensates for the loss of FP-derived Shh, as suggested by *Gli2* mutants (Danesin and Soula, 2017). Hence, No-derived Shh would follow a basal pathway. Consistently, abrogation of Shh in the mesoderm, performed at the same stage and for a similar duration, revealed a significant NT phenotype, even in the presence of both Shh-producing axial structures. Altogether, we propose that at least a significant fraction of Shh operating on the neuroepithelium stems from the No via a sclerotomal pathway that, at the stages examined, seems more active than the FP in promoting motoneuron development.

It is noteworthy that different procedures that perturb the production of Shh, or its signaling, have different effects on FP integrity. Whereas Gli2 mutants lack a FP (Matise et al., 1998), deletion of Shh in the FP does not affect its maintenance (Dessaud et al., 2010). In both chick and mouse, Shh was suggested to be necessary for initial FP induction but, later on, the FP becomes refractory to the ligand (Ribes et al., 2010). In our experiments, we used a membrane-tethered version of the high-affinity Shh inhibitor Hhip and also the Shh receptor Ptc1, with both resulting in the death of FP cells. This might be accounted for by a combination of ligand depletion with accumulation of Shh-Hhip or Shh-Ptc1 complexes at the cell membrane altogether compromising the epithelial integrity.

Furthermore, if Shh from the sclerotome is important for neuroepithelial development, is it possible to directly show its presence in this domain? Shh in the synthesizing cells of the FP and the No is intracellular and membrane bound (Chamberlain et al., 2008) (Fig. S2), whereas in the sclerotome it is expected to be extracellular and/or included in organelles (e.g. exosomes). Most protocols used for tissue processing will probably only keep the former type of immunoreactive protein and wash away the extracellular ligand in the sclerotome. Notably, when using a method that allowed proteoglycan/glycosaminoglycan preservation and/or perhaps also a different antibody, a previous study showed a sclerotomal localization of Shh protein (Gritli-Linde et al., 2001).

One open question is how Shh is transported through the sclerotome. Possible models could involve packaging of the ligand in No-derived exosomes (Vyas et al., 2014), diffusion of Shh released by matrix metalloproteinases in a lipid-free form (Dierker et al., 2009), secretion as multimeric complexes (Chen et al., 2004) and/or via carrier-mediated transport through the extracellular space (Parchure et al., 2018). The precise mechanism responsible for Shh transport in the present context remains to be unraveled.

If supplied to the NT from the sclerotome, it is inferred that neuroepithelial cells sense Shh from their basal pole that faces the mesoderm. Consistently, grafting No fragments in a basal, but not an apical, position, with respect to the NT, profoundly affects NT shape, motoneuron differentiation and Pax7 expression. Indeed, during normal development, the No underlies the basal domain of the NT, further supporting the idea that No-derived Shh can only reach the NT via a basal route. In line with this, it was reported that lipidated Shh enters cells of the imaginal disc in *Drosophila* only through its basolateral surface (Callejo et al., 2006). A similar phenomenon was observed in high-density human gastruloids, which self-organize into an epithelium. In these cultures, cells were responsive to BMP4 or Activin ligands only when presented from the basal side (Etoc et al., 2016), suggesting that cell polarization controls the ligand response.

The above findings are interesting in light of the proposed concept that apically localized cilia serve as antennae to sense and transduce a Shh signal (Corbit et al., 2005; Singla and Reiter, 2006). We suggest that, initially, Shh is presented from the basal side of epithelial cells from which it may be transported to the apical domain where cilia are localized. As an apical presentation of the No and associated Shh did not result in a significant effect in our implant experiments, we propose that cilia act as transducers of a Shh signal, but not as the primary antennae sensing the ligand. This further suggests that basal reception followed by baso-apical transport might be necessary for the activity of Shh arriving at the cilia. Growing evidence suggests that cilia-independent Shh reception occurs through basally localized cytonemes (Gradilla et al., 2018). Likewise, in the retina neuroepithelium, Shh and its co-receptor Cdo colocalize at the basal side of the cells where filopodia-like structures are present (Cardozo et al., 2014). An extreme example is provided by some cell types in which Shh signaling takes place even in the absence of cilia (Gradilla et al., 2018).

In the NT, direct visualization of labeled Shh revealed that the ligand concentrates in association with the apically localized basal bodies from which cilia stem, while forming a dynamic gradient in the ventral NT (Chamberlain et al., 2008). In light of the present results, the above observed graded distribution of ligand could be explained as being the end point of a transport process in which the ligand begins at the No, travels through the sclerotome, where it forms a ventro-dorsal gradient, and then reaches the neuroepithelium through its basal side where it finally concentrates in the apical cilia (Fig. 9G). Possible mechanisms mediating such a baso-apical transport associated with possible biochemical changes of the protein to make it available to cilia for signaling, remain to be unraveled. A microtubular network spanning the extent of neuroepithelial cells could be involved in this process (Chamberlain et al., 2008).

An initial presentation of Shh from the basal side of an epithelium seems to be of general significance as basal grafting of a No also elicited robust *in situ* differentiation of DM progenitors into myocytes, when compared with an apical implant that exhibited only a mild phenotype. How can this differential effect be explained given that the endogenous ligand apparently arrives from the apical sclerotomal direction? Initially, pioneer myotomal cells in the epithelial somite face the No from their basal aspect (Kahane et al., 1998). Upon sclerotome dissociation, the epithelial DM is consolidated and becomes composed of a central sheet and four inwardly curved lips pointing towards the sclerotome. We and others showed that the four DM lips are the main sources of myotomal cells (Kalcheim et al., 1999), and it is their basal domain that points towards the sclerotome from which Shh arrives. Next, myotomal progenitors enter the nascent myotome and a basement membrane begins assembling between the myotome and sclerotome (Borycki, 2013). At this time, the central DM sheet also contributes myotomal progenitors by direct translocation (Ben-Yair et al., 2011), which then differentiate in a Shh-dependent manner (Kahane et al., 2013). These precursors exhibit an inverse apicobasal polarity when compared with central DM cells (Ben-Yair et al., 2011). Hence, both the DM lips and these translocating progenitors point their basal surfaces towards the source of endogenous Shh, are probably the main targets for Shh activity and also account for the partial effect of the apical grafts.

As discussed above, the basal domain of the DM/myotome and the NT epithelia are characterized by the presence of a surrounding basement membrane. An association between Shh and the basal lamina has been shown. In cerebellar granule precursors, the laminin-containing basement membrane binds and enhances Shh signaling (Blaess et al., 2004). Similarly, Shh induces the activation of Myf5 in the mouse DM. Myf5^+^ cells then translocate to the myotome and upregulate α6β1 integrin and dystroglycan, allowing a myotomal basement membrane to be assembled (Borycki, 2013). This is further confirmed in Shh-deficient mice, which fail to form a myotomal basement membrane and in which myotomal differentiation is delayed (Anderson et al., 2009; Borycki et al., 1999). Additionally, Shh immunoreactive protein was found to localize in the basement membrane surrounding the NT (Gritli-Linde et al., 2001). Taken together, these results suggest that, in both the NT and the DM/myotome, a feed-forward mechanism may exist whereby Shh controls laminin expression and basement membrane assembly. This could allow a local concentration of the ligand and/or signal stabilization. However, the basement membrane alone is unlikely to serve as the common pool, as sequestering the ligand specifically in the sclerotome by membrane-associated Hhip:CD4 was highly effective. In addition, others have shown that the ligand is present in the mesoderm itself (Gritli-Linde et al., 2001).

Our present findings support the importance of NT-somite interactions that are pivotal for the normal patterning of trunk components. For example, opposite gradients of retinoic acid and Fgf8 in mesoderm are required for NT development (Diez del Corral et al., 2003). In addition, the nascent DM controls the timing of neural crest delamination by modulating noggin mRNA and BMP activity in the NT (Sela-Donenfeld and Kalcheim, 2000). Reciprocally, Bmp4 and/or Wnt1 from the NT pattern somite-derived myogenesis (Abu-Elmagd et al., 2010; Marcelle et al., 1997). The formation of the dorsal dermis is influenced by NT-derived Wnt1 and by neurotrophin 3 (Brill et al., 1995; Marcelle et al., 1997; Sela-Donenfeld and Kalcheim, 2002). Furthermore, interactions between neural and somitic cells control neural crest migration, and segmentation of peripheral ganglia and nerves, as well as specific aspects of myogenesis (Kalcheim, 2011; Kalcheim and Goldstein, 1991). The present results raise the intriguing possibility that the dual activity on both motoneurons and the myotome of Shh released into the sclerotome serves to couple and coordinate development of the neuromuscular system.

## MATERIALS AND METHODS

### Embryos

Chick (*Gallus gallus*) and quail (*Coturnix japonica*) eggs were from commercial sources (Moshav Orot and Moshav Mata, respectively). All experiments were performed using quails except for those involving *in situ* hybridizations, which were performed in chick embryos.

### Expression vectors and electroporation

Expression vectors used were: pCAGGS-GFP, pCAGGS-RFP (Krispin et al., 2010), Ptc1 (Briscoe et al., 2001), PTC^Δloop2^ (Briscoe et al., 2001; Kahane et al., 2013), a retinoic acid reporter fused to alkaline phosphatase (pRARE-AP, from J. Sen) (Gupta and Sen, 2015), a dominant-negative pan-retinoic acid receptor (RAR403dn-IRES-GFP, from S. Sockanathan) that abrogates retinoic acid signaling (Novitch et al., 2003), full-length Shh (Kahane et al., 2013), cholesterol-deficient Shh (mShh-N-YFP, from V. Wallace) (Beug et al., 2011) that was subcloned into pCAGGS, and mHhip1 (Kahane et al., 2013). To produce Hhip1:CD4, a membrane-tethered version of Hhip1, the transmembrane and intracellular domains of mouse CD4 were fused to the C-terminal domain of Hhip1 lacking amino acids A679 to V700, as described previously (Holtz et al., 2015; Kwong et al., 2014), and further subcloned into pCAGGS for electroporation.

For electroporations, DNA (1-4 µg/µl) was microinjected into the center of flank-level epithelial somites (somites 20-25) of 23-25 somite-stage embryos. Electroporations were performed at the ventral half of epithelial somites (prospective sclerotome). To this end, the positive tungsten electrode was placed under the blastoderm in a location corresponding to the ventro-medial region of epithelial somites for a length of about seven segments; the negative electrode was placed in a superficial dorso-lateral position, with respect to the same somites (Ben-Yair et al., 2011; Halperin-Barlev and Kalcheim, 2011; Kahane et al., 2007, 2013).

For hemi-NT electroporations, DNA was microinjected into the lumen of the NT. One tungsten electrode was placed underneath the blastoderm on one side of the embryo and the other electrode was placed in a superficial position at the contralateral side. For FP electroporations, the positive electrode was inserted under the blastoderm near the midline and the negative electrode was placed over the dorsal NT. In some cases, double electroporations to the hemi-NT and the sclerotome were performed sequentially. An ECM 830 square wave electroporator (BTX) was used. One 12 V pulse was applied for 5 ms.

### No grafts

No fragments comprising a length of seven or eight segments were enzymatically excised from donor embryos with 25 somite pairs as described previously (Charrier et al., 2001) and kept in ice-cold PBS until grafting. For grafting at the apical side of the NT, the ectoderm and dorsal NT of host embryos were cut, and the No piece was placed in the NT lumen. Subsequently, 1 day after implantation, the grafts were usually found in the dorsal region of the NT facing its cavity. For basal grafting with respect to the NT, a slit was made between the somites and the NT, and the No fragments were inserted. A similar, but more profound slit, was made so that the ventral sclerotome abutting the apical side of the DM could be reached. To reach the basal domain of the DM, the ectoderm was cut to precisely accommodate the length of the No fragment. Following microsurgery, embryos were reincubated for an additional 24 h.

### Immunohistochemistry

Embryos were fixed overnight at 4°C with 4% formaldehyde in PBS (pH 7.4) followed by washes in PBS. Most immunostainings, except for desmin, were performed on whole-embryo fragments. Immunolabeling for desmin was performed on tissue sections, as described previously (Burstyn-Cohen and Kalcheim, 2002; Kahane et al., 2001).

For whole-mount immunostaining, antibodies were diluted in PBS containing 1% Triton X-100 and 5% newborn calf serum, and tissues were incubated overnight at 4°C on a rotary shaker. Next, the samples were washed twice in a large volume of PBS/1% Triton X-100, the first time for 10 min and the second time for 2 h at room temperature. Secondary antibodies were similarly diluted in PBS/1% Triton X-100/5% newborn calf serum and incubated overnight followed by repetitive washes. Embryo fragments were dehydrated in increasing ethanol solutions (30%, 70%, 90% and 100%, 10 min each) followed by toluene (two times, 10 min each), then embedded in paraffin wax and sectioned at 8 µm. Paraffin was removed in Xylene and slides were rehydrated in decreasing ethanol solutions.

The following antibodies were used: rabbit anti-GFP (1:2000, Invitrogen, Thermo Fisher Scientific, A6455) and mouse anti-desmin (1:200, Molecular Probes, 10519). Monoclonal antibodies against Pax7 (PAX7-s, 1:20), Shh (5E1, 1:20) and Hb9 (1:200) were obtained from the Developmental Studies Hybridoma Bank (University of Iowa). Phosphorylated Smad 1-5-8 (PSmad, 1:1000) was a gift from Ed Laufer (Columbia University, New York, NY, USA). Anti-Histone H3 (phospho S10) was obtained from Abcam (1:500, 14955) and anti-caspase 3 (1:200, ab 32351) was obtained from Cell Signaling Technology. Detection of DNA fragmentation was carried out using TUNEL staining (ab66110, Abcam) according to the manufacturer's instructions. Nuclei were visualized with Hoechst stain (Abcam, 33342).

### *In situ* hybridization

Embryos were fixed in Fornoy (60% ethanol, 30% formaldehyde, 10% acetic acid), then dehydrated in ethanol/toluene, processed for paraffin wax embedding and sectioned (10 μm). Slides were rehydrated in toluene/ethanol/PBS, treated with proteinase K (1 µg/ml, Sigma-Aldrich, P2308) at 37°C for 7 min, and then fixed in 4% formaldehyde at room temperature for 20 min. Next, slides were washed in PBS followed by 2× SSC and hybridized in hybridization buffer [1× salt solution composed of 2 M NaCl, 0.12 M Tris, 0.04 M NaH_2_PO_4_2H_2_O, 0.05 M Na_2_HPO_4_, 0.05 M EDTA (pH 7.5), 50% formamide, 10% dextran sulfate, 1 mg/ml yeast RNA, 1× Denhardt solution] containing 1 µg/ml DIG-labeled RNA probes (prepared with a DIG RNA labeling mix, Roche, 11277073910) overnight at 65°C in a humidity chamber. Post-hybridization, slides were rinsed in a rotating incubator with 50% formamide and 1× SSC, 0.1% Tween 20 until coverslips dropped, followed by two washes in MABT [10% Maleic acid 1 M (pH 7.5), 3% NaCl 5 M, 0.1% Tween 20] and preincubation in MABT/2.5% fetal calf serum (FCS). Anti-DIG-AP antibody (1:1000, Roche, 11093274910), diluted in MABT/2% BBR/20% FCS, was then added before overnight incubation at room temperature. This was followed by rinsing in MABT and then in NTMT [2% NaCl 5 M, 10% Tris HCl 1 M (pH 9.5), 5% MgCl_2_ 1 M, 0.1% Tween 20], followed by incubation in NTMT/1:200 NBT/BCIP Stock Solution (Sigma-Aldrich, 11681451001) at 37°C until the alkaline phosphatase reaction was completed. The following probes were used: *Hhip1* (from J. Briscoe, Crick Institute, London, UK), *Nkx*2.2, *Nkx 6.1*, *Nkx* 6.2, *Olig2*, *Ptc2* (from J. Ericson, Karolinska Institute, Stockholm, Sweden), and *Gli1* and *Gli3* (from A. G. Borycki, University of Sheffield, Sheffield, UK).

### Data analysis and statistics

Four to 26 embryos were analyzed per experimental treatment. Each experiment was repeated at least three to five times. The number of Hb9^+^ motoneurons was counted in five to ten alternate sections per embryo. The average number of Hb9 cells counted per embryo was 650, ranging between 200 to 1400 cells. The number of phospho-histone H3 (pH3) or caspase 3^+^ nuclei was monitored in seven to ten sections per embryo in four to eight embryos per treatment. The average number of pH3 cells counted per embryo was 150, ranging from 53 to 240 cells. Caspase quantifications are in Results.

Myotomes were defined using desmin staining. The area occupied by desmin^+^ myotomes was measured in alternate sections of three to 16 embryos per experimental treatment. Sclerotomes of four to six embryos were defined in the mediolateral aspect as the tissue between the myotome and the NT, and in the dorsoventral extent as the mesenchyme between the dorsomedial lip of the DM up to the dorsal border of the cardinal vein and aorta. The surface area of hemi-NTs was recorded in four sections per embryo. Myotomal, sclerotomal and hemi-NT areas, as well as the area and intensity of cells expressing the p-Smad 1,5,8 or pRARE-AP, were measured using ImageJ software (National Institutes of Health). All results are expressed as the mean proportion of positive cells or area in treated contralateral sides compared with control contralateral sides or with control GFP ±s.e.m.

In a control experiment, we tested whether area measurements are a faithful representation of cell number. To this end, we compared the number of Hoechst^+^ nuclei in hemi-NTs electroporated with Hhip:CD4/contralateral side versus control GFP/contralateral side, and found them to be not significantly different from the equivalent ratio of surface area. Consequently, the calculated ratio of area to cell number was similar (1.03±0.032, *n*=6 embryos sectioned at 5 µM, five sections/embryo). Thus, surface area was validated as a reliable measure of overall cell number.

Images were photographed using a DP73 cooled CCD digital camera (Olympus) mounted on a BX51 microscope (Olympus) with Uplan FL-N 20×/0.5 and 40×/0.75 dry objectives (Olympus). For quantification, images of control and treated sections were photographed under the same conditions. For figure preparation, images were exported into Photoshop CS6 (Adobe). If necessary, the levels of brightness and contrast were adjusted to the entire image and images were cropped without color correction adjustments or γ adjustments. Final figures were prepared using Photoshop CS6.

The significance of results was determined using the non-parametric Mann–Whitney test. All tests applied were two-tailed, and a *P*-value of 0.05 or less was considered statistically significant. Data were analyzed using the IBM SPSS software version 25. The number of embryos analyzed for each treatment (*n*) is detailed in the Results section. *P*-values can be found both in the Results section and in the corresponding figure legends.

## Supplementary Material

Supplementary information
