# Supplementary material for: Neural tube development depends on notochord-derived sonic hedgehog released into the sclerotome
Source: Development. 2020 May 26;147(10):dev183996. doi: 10.1242/dev.183996 (PMC7272346; doi:10.1242/dev.183996)
Supplement: Supplementary information [file develop-147-183996-s1.pdf]

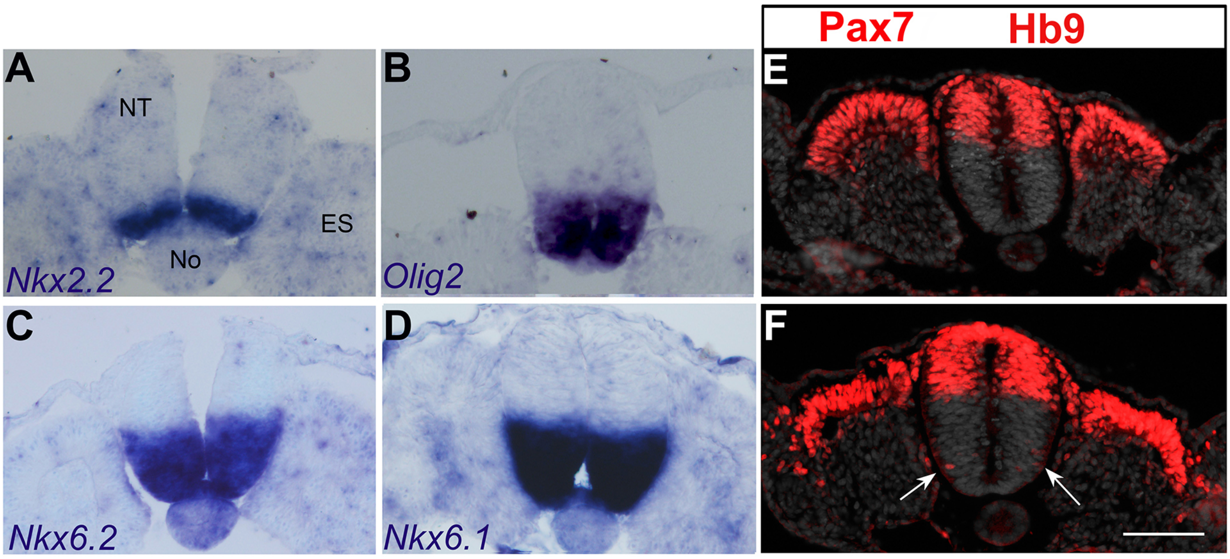

Fig. S1- Expression of neural tube markers by the time of *in ovo* electroporation

(A-D) In situ hybridization of embryos aged 25 somite pairs showing expression of ventral neural tube (NT) markers at the epithelial somite (ES) stage.

(E,F) Co-immunolabeling of Pax7 and Hb9 antibodies. Note in E that only Pax7 is expressed in both dorsal NT and dorsal aspect of the ES, whereas the first Hb9+ ventral motoneurons appear at a later stage following somite dissociation (arrows in F). Both dorsal Pax7 and ventral Hb9 immunostainings are in red as the respective antibodies are monoclonal of the same subtype. Abbreviations, No, notochord. Bar=50µM.

Development • Supplementary information

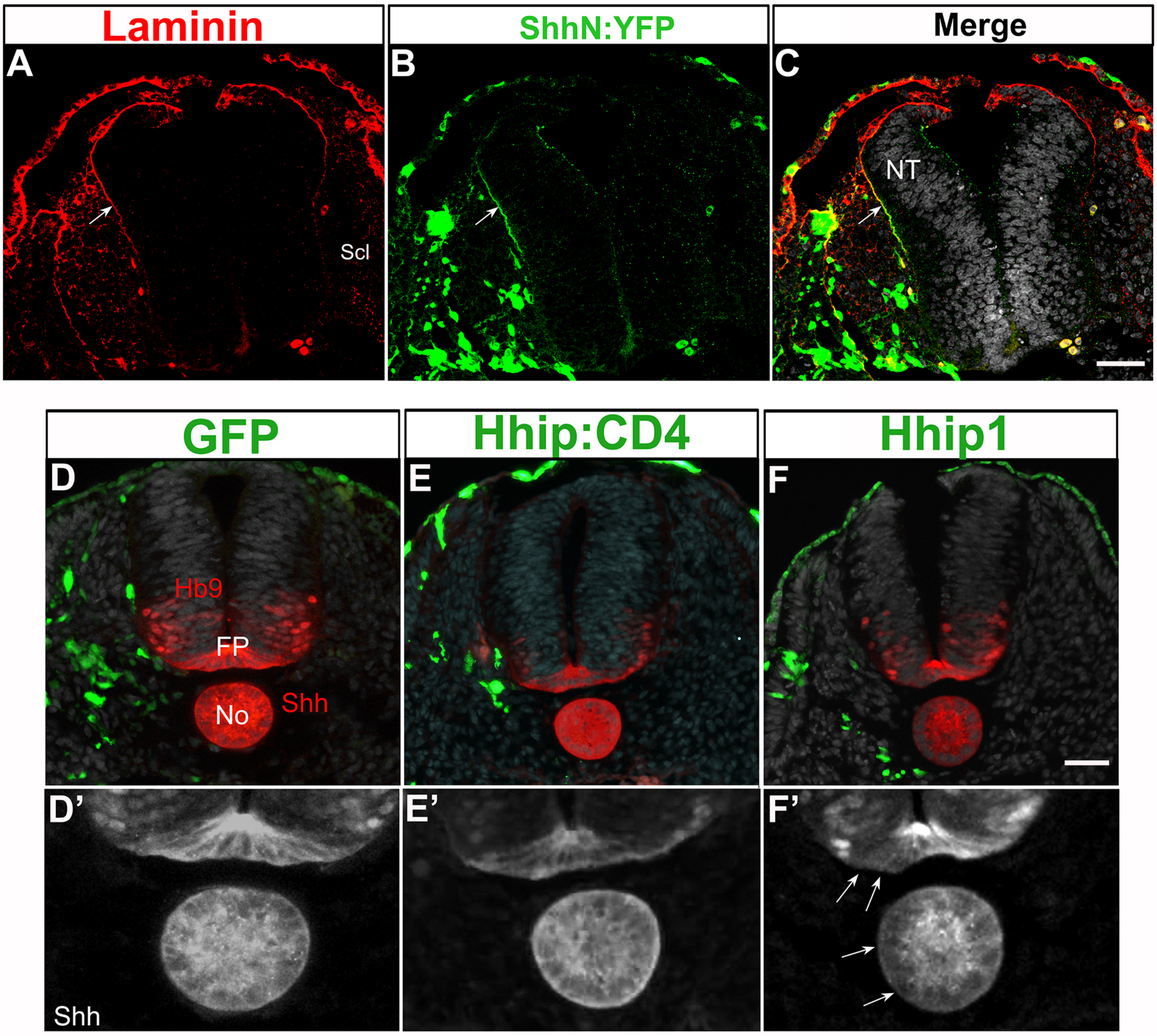

Fig. S2- Differential behavior of secreted Hhip1 compared to membrane-tethered Hhip:CD4

(A-C) ShhN:YFP, electroporated into sclerotome (Scl, green), colocalizes with laminin (red) in the basement membrane around the neural tube (NT) (arrows).

(D-F') Immunolabeling with Shh and Hb9 antibodies of embryos electroporated with control GFP (D,D'), Hhip:CD4 (E,E') or Hhip1 (F,F'). Note that Hhip1 caused a reduction in ipsilateral Shh in both notochord (No) and FP (FP) (arrows in F') whereas GFP or Hhip:CD4 were without effect. Bars=50 µM.

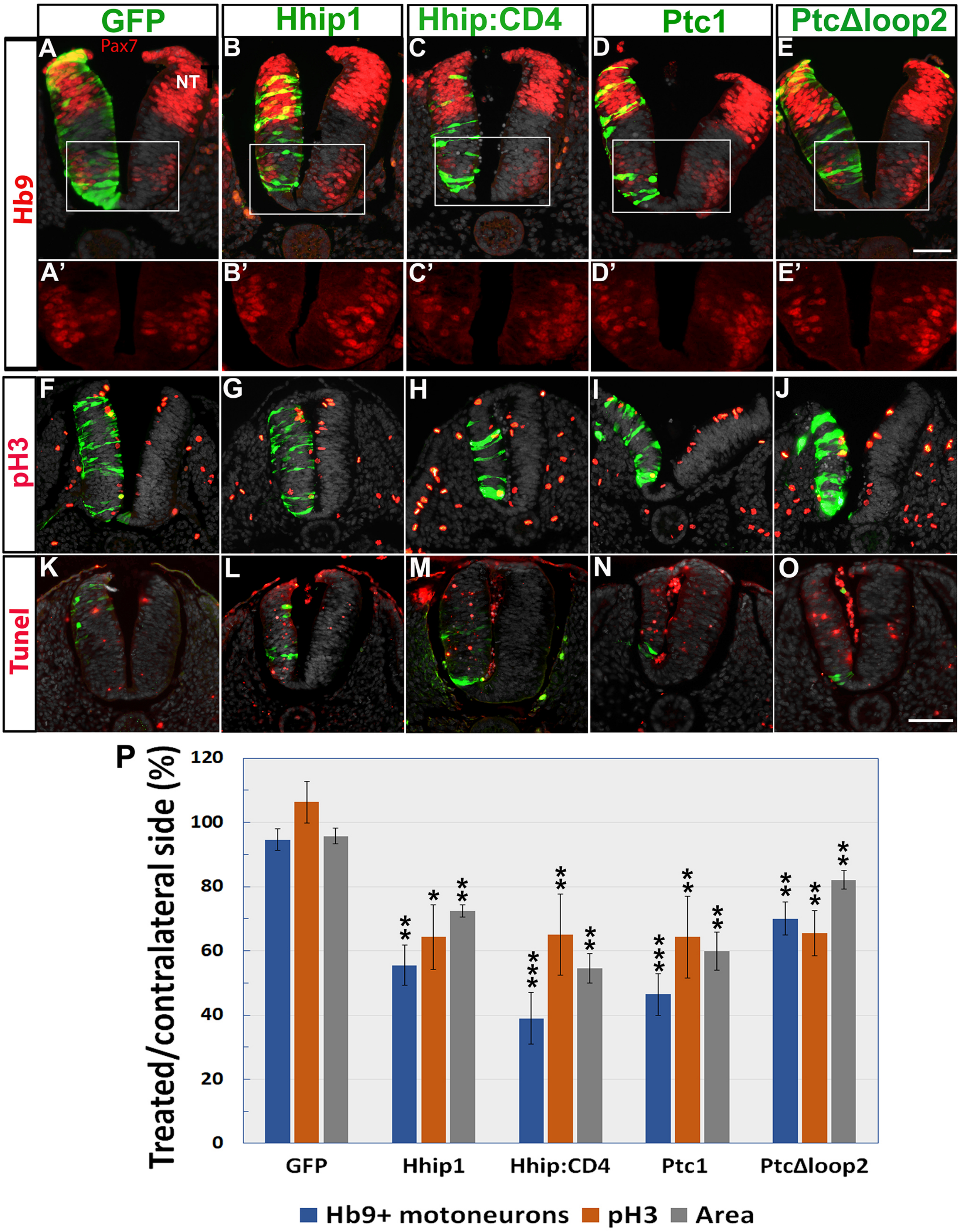
GFP
Hhip1
Hhip:CD4
Ptc1
PtcΔloop2
Hb9
pH3
Tunel
A
Pax7
NT
B
C
D
E
A'
B'
C'
D'
E'
F
G
H
I
J
K
L
M
N
O
P
Treated/contralateral side (%)
120
100
80
60
40
20
0
GFP
Hhip1
Hhip:CD4
Ptc1
PtcΔloop2
Hb9+ motoneurons
pH3
Area

Fig.S3- Depletion of Shh in the NT by Hhip:CD4 phenocopy those exerted by Hhip, Ptc1 and PTC$^{\Delta loop2}$

(A-E') Electroporation of depicted plasmids (green) to hemi-NTs followed a day later by immunostaining for Pax7 and Hb9. A'-E' represent higher magnifications of the boxed regions in A-E.

(F-J) staining of mitotic nuclei with anti pH3 (red) following electroporation with depicted plasmids.

(K-O) Tunel staining (red). Enhanced apoptosis is seen in the hemi-NTs adjacent to all electroporations when compared to the respective contralateral sides or to control GFP. Note weak immunostaining of electroporated plasmids (green) because no anti-GFP antibodies were implemented to enable better visualization of apoptotic nuclei.

(P) Quantification of motoneurons, mitotic nuclei and area. *$p<0.05$, **$p<0.03$, ***$p<0.01$. Bar=50μM.

Development • Supplementary information

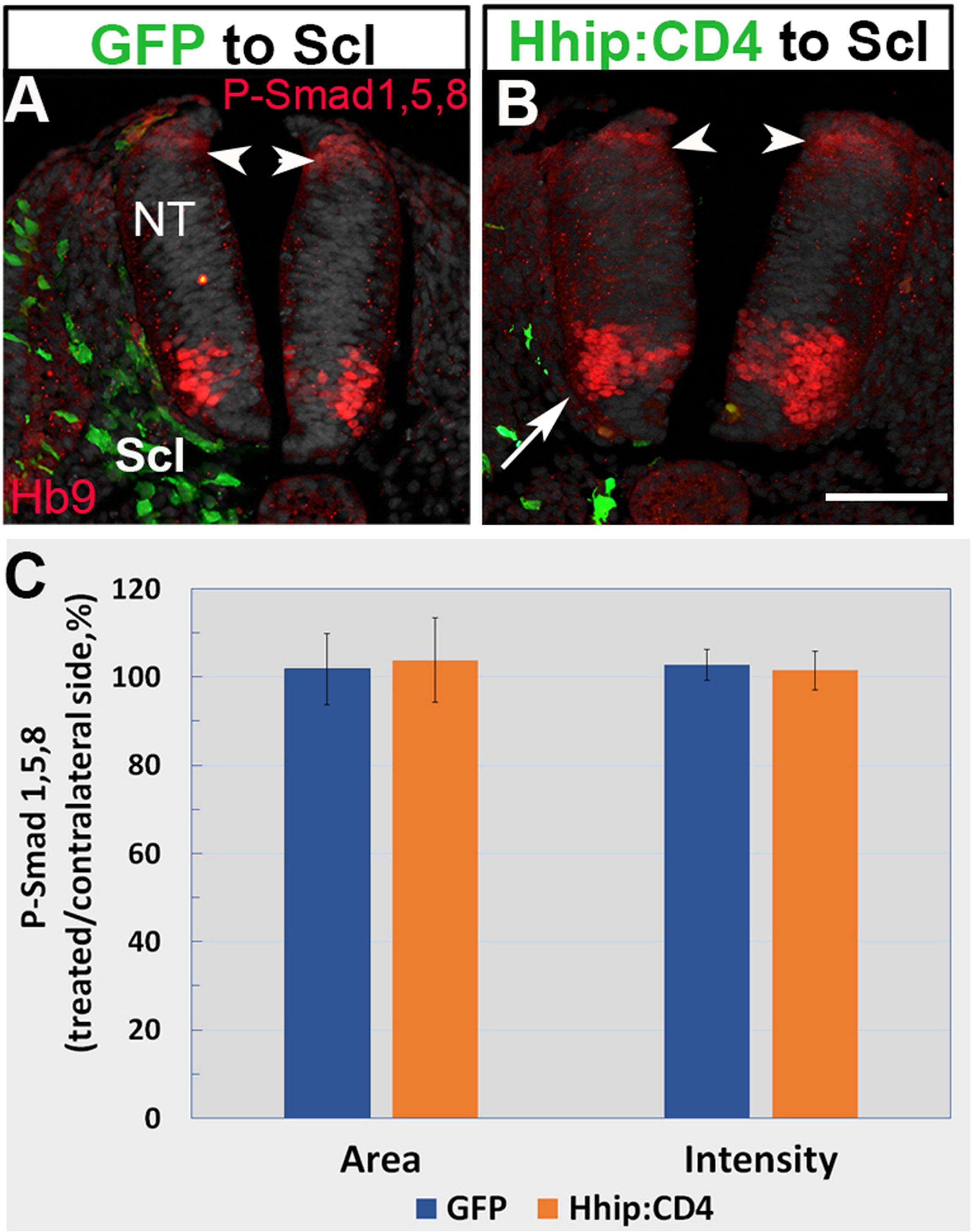

Fig. S4- Electroporation of Hhip:CD4 to sclerotome has no effect on BMP signaling in dorsal NT.

(A,B) Missexpression of Hhip:CD4 in sclerotome (green) had no effect on expression of phospho-Smad 1,5,8 in the dorsal NT compared to control GFP (arrowheads), yet reduced the number of Hb9+ motoneurons (arrow in B). (C) Quantification of the area and intensity of phospho-Smad 1,5,8 expression. Bar=50 μM.

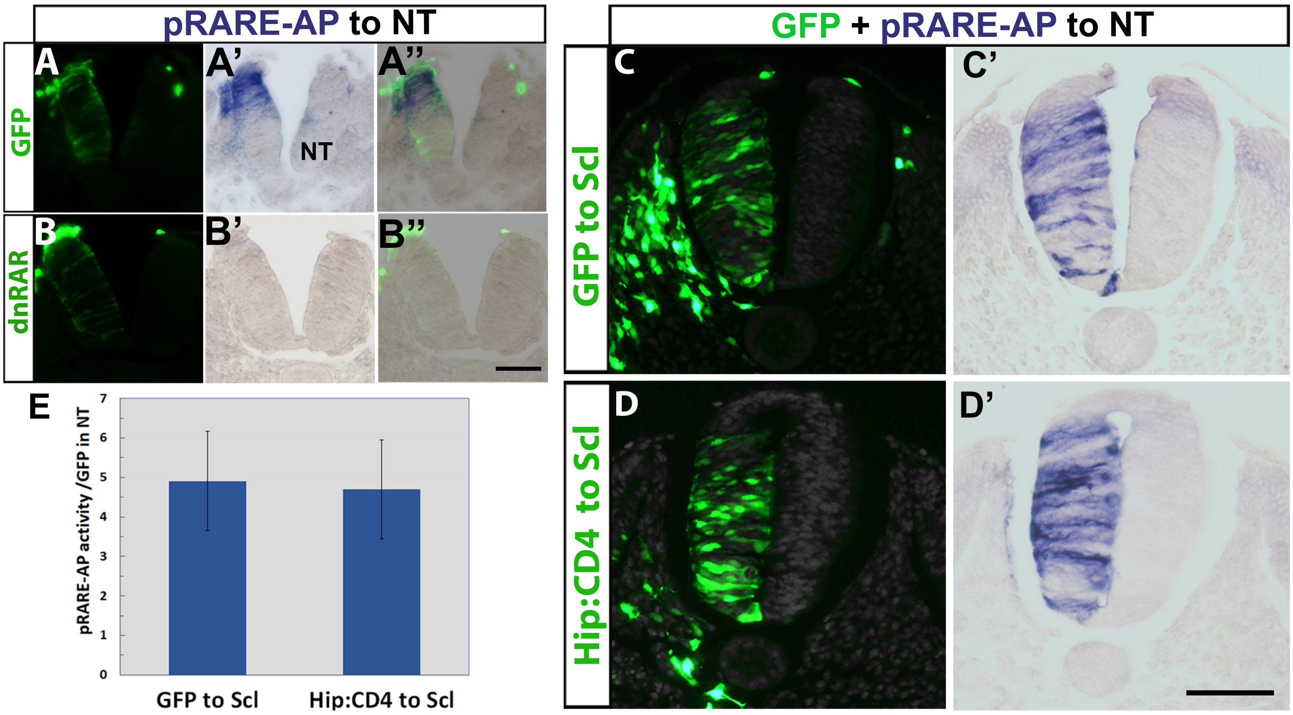

Fig. S5- Inhibition of Shh activity in sclerotome has no effect on retinoic acid signaling in the neural tube.

(A-B”) Specificity of the retinoic acid reporter (pRARE-AP). Note in A-A” the expression of pRARE-AP in control GFP-electroporated NT. In contrast, no signal is apparent when retinoic acid activity is abolished by a dnRAR plasmid (B-B”). A” and B” are overlays of the precedent panels, respectively.

(C-D’) Double electroporations of control GFP or Hhip:CD4 to the sclerotome (C and D, respectively) and co-electroporation of RARE-AP together with GFP to the NT of the same embryos (C,C’, D,D’).

(E) Data quantification. Missexpression of Hhip:CD4 in sclerotome had no effect on RARE-AP/GFP activity in NT. Results represent mean values of RARE-AP/GFP±SEM. Bars=50 µM.

Development • Supplementary information

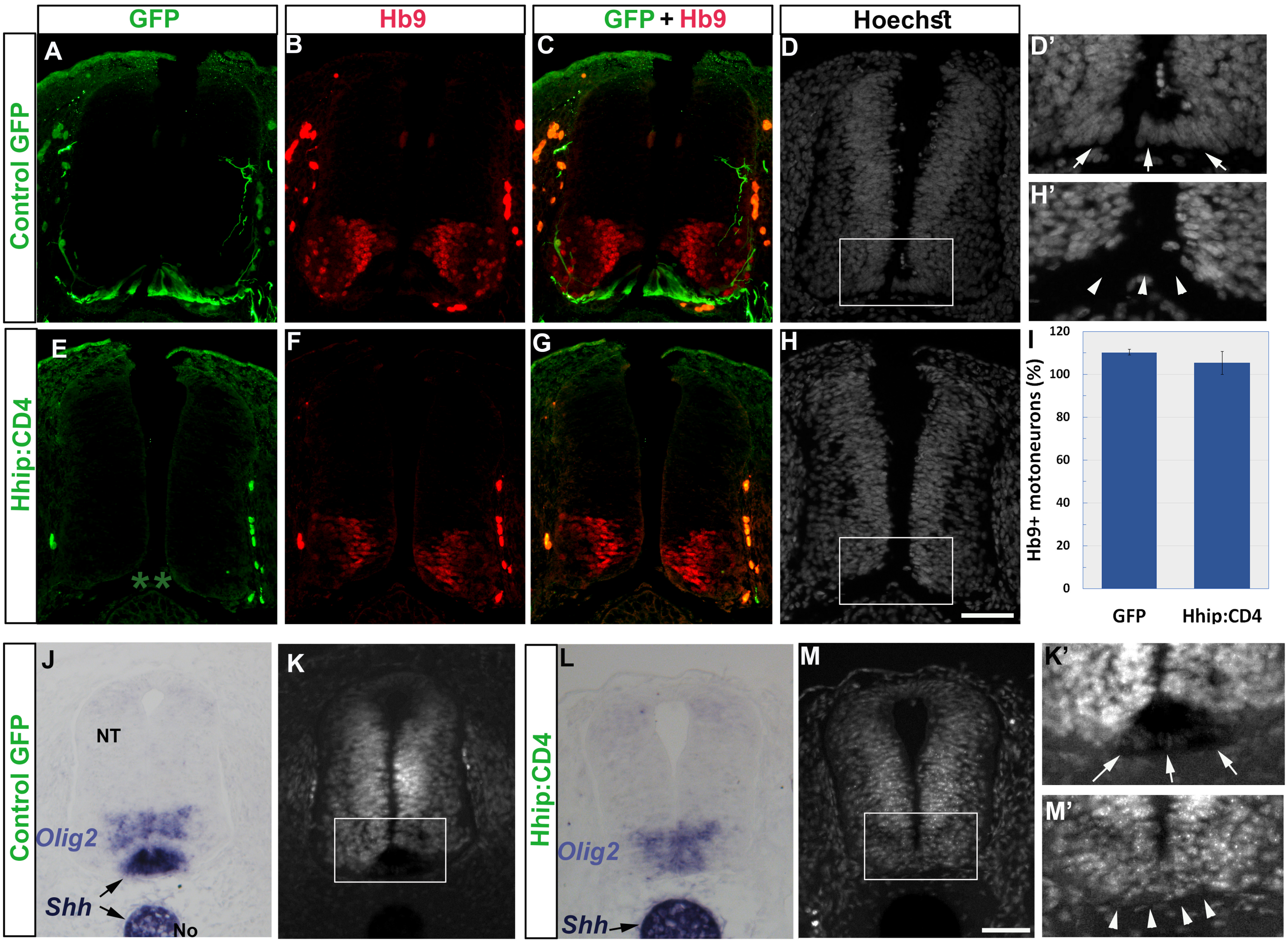

Figure S6- Deletion of the FP has no significant effect on Hb9+ motoneurons or *Olig2+* precursors

(A-D') Dorsoventral electroporation of control GFP showing in A, the presence of the labeled floor plate (FP). In B, note Hb9+ motoneurons dorsal to the labeled FP. (C) Overlay of A and B. (D, D') Hoechst nuclear stain. Note in the higher magnification (D') the basal localization of nuclei (arrows).

(E-H') Loss of FP tissue 40 hours post-Hhip:CD4 electroporation as marked by absence of GFP. Asterisks (**) denote absence of a FP. Arrowheads in H' mark an open ventral NT lacking FP cells. (I) Quantification showing no effect of FP deletion on the proportion of Hb9+ motoneurons in caudal brachial (electroporated)/rostral brachial (intact region).

Development • Supplementary information

(J-M') Double in situ hybridization for *Olig2* (light blue) and *Shh* (dark blue) of control GFP (J,K,K') or Hhip:CD4 electroporated embryos (L, M,M'). Note that in controls, *Olig2* (light blue) is dorsal to *Shh* mRNA expression (intense blue in FP and also seen in No). Between them, a characteristic gap corresponding to ventral interneurons is apparent. In contrast, upon electroporation of Hhip:CD4, the FP, with its typical basal nuclei is lost (arrows in D',K' compared to arowheads in M'), and a concomitant disappearance of *Shh* mRNA signal is apparent. In spite of that, *Olig2* expression seems unaffected (L). Abbreviations, No, notochord; NT, neural tube. Bars=50 µM.

Development • Supplementary information
